# Biofilms: Formation, drug resistance and alternatives to conventional approaches

**DOI:** 10.3934/microbiol.2022019

**Published:** 2022-07-04

**Authors:** Ruba Mirghani, Tania Saba, Hebba Khaliq, Jennifer Mitchell, Lan Do, Liz Chambi, Kelly Diaz, Taylor Kennedy, Katia Alkassab, Thuhue Huynh, Mohamed Elmi, Jennifer Martinez, Suad Sawan, Girdhari Rijal

**Affiliations:** 1 Equal authorships; 2 Department of Medical Laboratory Sciences, Public Health & Nutrition Sciences, College of Health Sciences and Human Resources, Tarleton State University, a Member of Texas A & M University System, Fort Worth, TX 76104, USA

**Keywords:** biofilms, bacterial resistance, prevention, prophylaxis, probiotics

## Abstract

Biofilms are aggregates of bacteria, in most cases, which are resistant usually to broad-spectrum antibiotics in their typical concentrations or even in higher doses. A trend of increasing multi-drug resistance in biofilms, which are responsible for emerging life-threatening nosocomial infections, is becoming a serious problem. Biofilms, however, are at various sensitivity levels to environmental factors and are versatile in infectivity depending on virulence factors. This review presents the fundamental information about biofilms: formation, antibiotic resistance, impacts on public health and alternatives to conventional approaches. Novel developments in micro-biosystems that help reveal the new treatment tools by sensing and characterization of biofilms will also be discussed. Understanding the formation, structure, physiology and properties of biofilms better helps eliminate them by the usage of appropriate antibiotics or their control by novel therapy approaches, such as anti-biofilm molecules, effective gene editing, drug-delivery systems and probiotics.

## 1. Introduction

Biofilms are networks of aggregated living microorganisms that are formed either on biotic or abiotic moist surfaces [1],[2]. They are well-known predominating micro-ecosystems which are not only distributed widely in most natural environments but can be adapted to survive in extreme environmental conditions, such as UV radiation, high salinity, poor nutrients, high temperature, high pH and antibiotics [3],[4]. Because of their power of adaptation in extreme conditions, they are assumed to have developed self-protective mechanisms, creating an environment for their growth and survival, in addition to enhancing the communications among the microcolonies within the biofilms [3],[5]. Three-dimensional structure of biofilms is established by the layer which is formed by the aggregation and composition of the secretory insoluble substances produced by the associated microorganisms. The layer made of extracellular substances is collectively known as the extracellular polymeric substance (EPS), which has an indispensable role in the formation of biofilms [3],[6]. EPS also supports the transformation of biofilms from reversible to irreversible and establishes the biofilm on a suitable surface [7],[8]. Thus, the slimy EPS not only helps organisms adhere to each other but also protects biofilms from any adverse synthetic reactions [8],[9]. Biofilms are diverse, depending on organism types and environmental factors [9]. Most of the biofilms are aggregations of similar or different bacterial species, but they can also be composed of other organisms, such as fungi, algae, or protozoa [9]–[11]. They are usually tangled as a mass of prolonged and infectious organisms, along with their capacity to guard themselves against various antimicrobial drugs. Sugars, proteins and nucleic acids present in the EPS are used by some organisms for their metabolism, and their metabolic end products serve as the nutritional sources for other types of organisms living in the proximity bounded by the EPS [12],[13]. In addition, EPS supports cell-cell and cell-matrix communications, creating the synergistic interactions. The extracellular matrices of the biofilms give protection to the microbes from different factors in their surrounding environment [14],[15]. EPS, therefore, has a significant role in biofilm formation and its protection besides limiting the diffusion of antibiotics [16]. Interestingly, not all the organisms can be a part of the biofilm; only organisms which favor the association with other organisms are a part of a biofilm [2],[17]. The main function of the biofilms is the support for the propagation of diverse microorganisms and their survival [3],[14]. The biological and environmental factors in biofilms for microorganisms are substantially different from those in planktonic environment [18].

## 2. Biofilm formation

Biofilm formation is a dynamic complex process that constitutes a multifaceted nature [2],[19],[20], and it is triggered, modulated and dispersed by the microorganisms themselves which are associated with and participate in a biofilm community [3]. The planktonic phenotype of the microorganism, which appears during initial colonization on a new surface, is considered as the floating or the drifting type of microorganism that can be moved to new habitats at various depths. It can adhere to the surface, populate and become a sessile biofilm, depending on the environmental factors [21]. A sessile community is permanently or covalently bonded to a surface, forming a matrix phenotype or a buoyant. The acquired pellicle, however, is a unique first stage in biofilm formation on an aqueous surface which provides an ideal growth environment, supporting rapid biofilm growth within minutes, such as the pellicle formed closed to the enamel in the oral cavity [2],[22].

**Figure 1.**
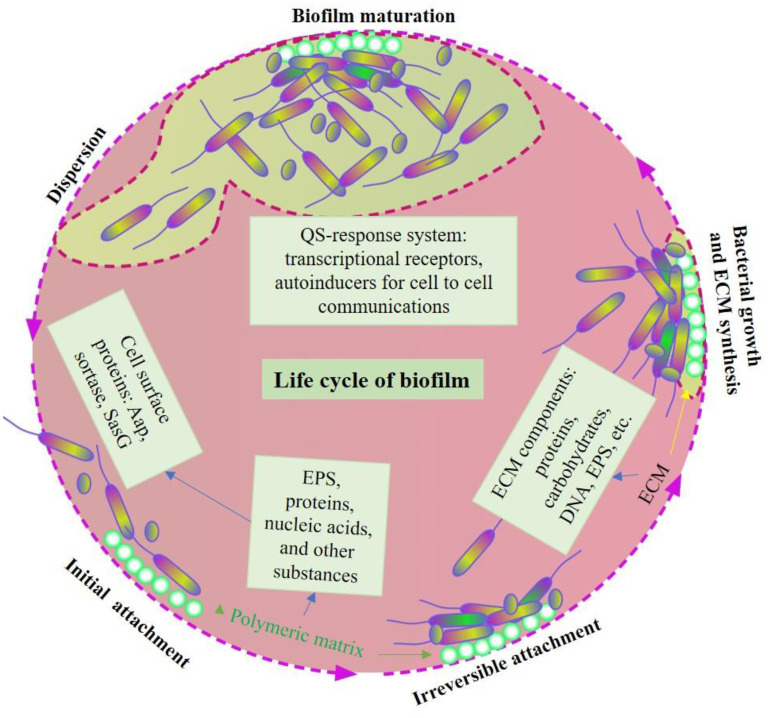
Formation, maturation and dispersion of biofilm.

Regarding bacterial involvement, both Gram-positive and Gram-negative bacteria can form biofilms, such as *Staphylococcus* and *Pseudomonas* species. Formation of biofilm starts after some free moving microorganisms come into contact with a suitable surface and establish the communication roots to gather sources of nutrients and the production of a slimy material known as EPS [17],[23]. Overall, biofilm formation has five steps: 1) initial reversible attachment between the bacteria and the friendly surface; 2) irreversible attachment that leads to the formation of monolayer surface of extracellular matrix (ECM) comprising proteins, cellular debris, nucleic acids and polysaccharides; 3) maturation stage I, exhibiting biofilm growth and quorum sensing (QS); 4) maturation stage II, leading to formation of EPS to encase the biofilm and helping with cell-cell and cell-matrix interactions; and 5) dispersion stage, separating from parent biofilms and repeating the cycle in another place [3],[24]–[26]. (Figure 1).

Bacterial attachment on the abiotic or biotic surface starts the biofilm formation. Initial reversible attachment is converted to irreversible attachment by binding of bacteria to the surface by the polymeric matrix, which is made of various exoproteins and substances released by the bacteria themselves. For example, exopolysaccharide substances (EPSs), proteins, nucleic acids and other substances help bacterial adherence on the surfaces. In addition, certain bacterial surface proteins, such as Aap, sortase and SasG, also play vital roles in irreversible attachment. After irreversible attachment, bacteria start division with the release of various extracellular matrix proteins (ECMs) for biofilm growth. Proteins, carbohydrates, DNA and EPS are some major components of ECMs that support bacterial growth during biofilm extension. Various QS response systems, with the help of transcription receptors and autoinducers, support cell-to-cell communications during biofilm maturation. Finally, because of nutritional deficiency and over-population, bacterial colonies start dispersion from the matured biofilm to move to a different place, ultimately leading to the expansion of biofilm.

**Figure 2.**
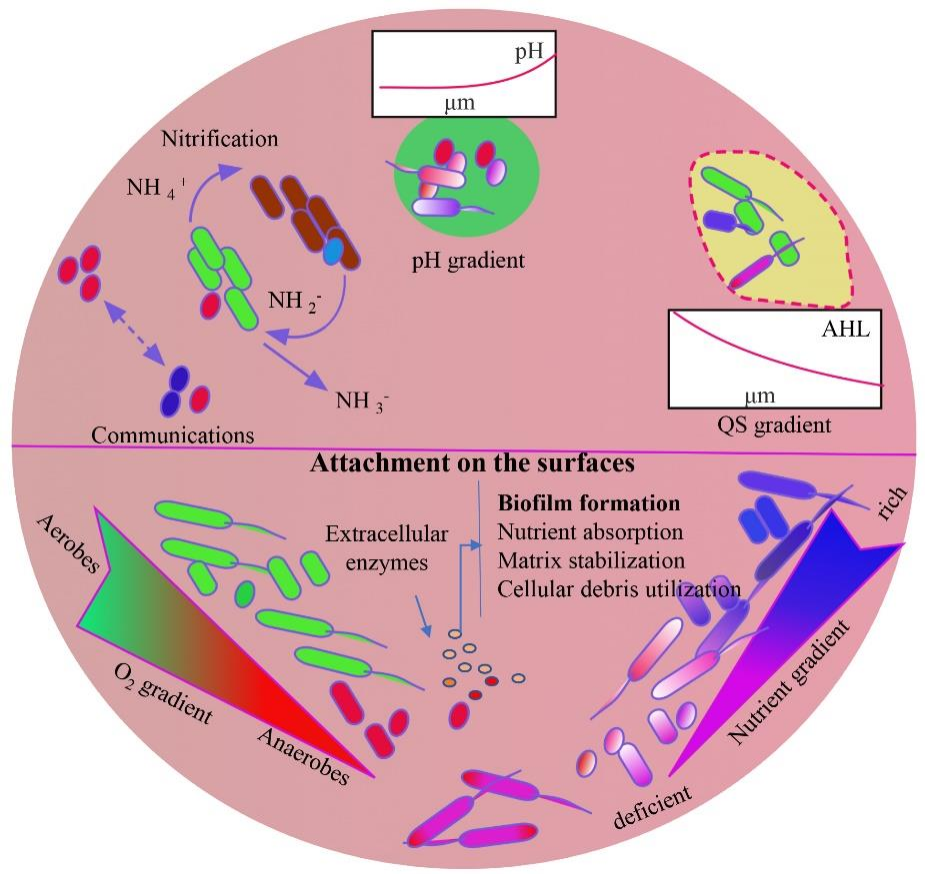
Biofilm microenvironment.

The detailed explanation of biofilm formation is beyond the scope of this paper. Briefly, bacteria must be close enough to the surface (biotic or abiotic) which allows the attractive and repulsive force that ultimately accepts the interactions with the organisms through various physical mediators. Bacterial mediators, such as flagella, support the initial interactions with the surface, and type IV pili help twitch, adhere and form microcolonies [27],[28] (Figures 1 and 2). Certain human pathogens, such as *S. epidermidis* and *S. aureus*, have adhesive genes that recognize the body surface (matrix proteins) for attachments. The number of adhesive genes is variable. For example, *S. aureus* has over 20 genes and *S. epidermidis* has only 6 genes [29],[30]. The initial reversible stage changes to the irreversible stage by the strong physical and chemical forces that lead to the formation and maturation of biofilms [31],[32]. Once cells attach irreversibly to the surface, they begin cell division to form microcolonies and secrete EPS, which helps biofilm formation [17],[33] (Figures 1 and 2). Once the first layer of biofilm has been established, cells from the same or different species participate to extend the biofilm further. Biofilm grows outward into a mushroom-like or tower-like formation [34],[35]. Participation of a new bacterial species or strains in the biofilm adds additional substances, such as polysaccharide biopolymers, DNA and proteins in the biofilm, forming complex EPS, and supports biofilm survival in adverse conditions [7],[8],[36].

Microorganisms are coordinated with each other and can share their individual survival skills, adding several protective advantages [3],[36]. Development of a biofilm community can be completed within hours, but its size depends on the microorganism types, source of nutrients and biotic or abiotic contact surfaces [17],[37]. Nutritional deficiency leads to dispersal of biofilm organisms to another location, which is known as a seeding dispersal for the search for nutrients [38]–[41]. Biofilm formation is thus a defensive mechanism of the bacteria in a stressful environment [36].

The alternative lifestyle of microorganisms in biofilm not only prolongs lifespan in diverse environments but enables them to linger and persist for a longer period. Anaerobic bacteria can eventually join the thicker biofilm, which has over 100 layers, by entering its deeper layer [42],[43]. Mature biofilms turn eventually into a bacterial community. Active dispersal is a critical step not only to start biofilm at new sites but also to prevent nutritional deficiency and over-population in former established sites [41]. Many other non-organisms associated factors, such as pH, temperature, ionic strength and nutritional availability, play significant roles in affecting the biofilm formation rate [2],[14],[36].

As stated above, biofilms may have single or multiple microbial species. A single species biofilm assumes a temporary lifestyle where each organism participates in a group facilitating survivability in adverse conditions. Multiple species biofilm, such as dental biofilm, may have over 500 bacterial taxa, creating resistance with longer survivability compared to single species biofilms [44]–[46]. Within the biofilms, bacteria are encased by a self- produced EPS, forming microcolonies. Studies demonstrate that microcolonies not only include the same or different bacterial species but also have different organisms, such as fungi [47],[48]. EPS also contains water that helps regulate the flow of the substances and nutrients and maintains the hydrophilic and hydrophobic natures within the biofilms [49],[50]. The water channels separate microcolonies from each other, providing the heterogenous layers of biofilms [2],[51]. In addition, the polysaccharides present in EPS contribute to the ionic bonding with minerals, such as calcium and magnesium [8],[52]. However, the polysaccharides of Gram-positive bacteria display cationic properties [53]. Biofilms show polymorphic appearances with different growth patterns based on the availability of nutrients. For example, microcolonies grow exponentially when glucose is at a high level, facilitating a thick biofilm [2],[54]. Nitrifying bacteria in biofilms also supports biofilm adhesion on positively charged surfaces and increases the biofilm thickness within a short time [55],[56] (Figure 2). Microcolonies indicate a significant growth; however, the biofilms become thinner when there are not sufficient nutrients in their microenvironment [2],[57]. Importantly, EPS arranges in such a way that can shield microcolonies against negative environmental disturbances, such as UV radiation, and against abrupt changes in certain microenvironmental conditions, such as pH [52],[58] (Figure 2).

Biofilm formation starts after attachment of bacteria on the surfaces of biotic or abiotic substances, where they start nutrient absorption, cellular debris utilization and matrix stabilization for biofilm maturation. Gradients of oxygen, pH, QS and nutrition determine the biofilm microenvironment that influences its maturity stages, size and depth. Therefore, biofilm can be a suitable environment for the growth of various types of microorganisms, such as aerobic to anaerobic, alkaliphilic to acidophilic and normal to nutrient-deficient bacteria. Versatile types of bacterial populations in a biofilm contribute to complexity of the ECM, which has various types of enzyme-digested end products of substances, supporting the survival of bacteria in adverse environmental conditions.

## 3. Biofilm complexity

Biofilm is a complex and dynamic matrix network made of combinations of exopolysaccharides, proteins, lipids and environmental DNA (eDNA) synthesized by different associated microorganisms [2],[59]. The rigidity and structure of ECM can vary within different microbial species, such as *Escherichia coli, Bacillus subtilis, Staphylococcus aureus* and *Vibrio cholerae* [2],[14],[60]. Complexity of biofilms can be studied at molecular levels and demonstrated by spectroscopy [61],[62]. For example, *E. coli*, a biofilm creator, has been studied in detail and is capable of synthesizing a variety of autotransporter adhesins at the beginning of biofilm formation [63],[64]. Antigen 43, an autotransporter adhesin, is encoded by the gene agn43 and has two subunits: α and β. The α subunit is on the superficial surface of the cell and connects the β subunit, an integral outer membrane protein [65],[66]. The L-shaped proteins of α subunits and helical structures of β subunits are stabilized by hydrogen bonds, forming salt bridges. They connect with each other through physio-mechanical processes that are established by self-association and self-aggregation of microbes. A major component of *E. coli* biofilms is curli fibers [67],[68]. These fibers have two components, CsgB and CsgA, which are encoded by csgBAC [68]. The two major accessory proteins (CsgE and CsgF), and two components (CsgD and CsgG) are produced by csgDEFD. CsgD regulates cellulose and curli fibers, and CsgG plays a role in CsgA translocation [68]. The curli fibers are abundant in a β-sheet structure. The resulting protein network provides integrity of a biofilm, resisting denaturation. Cellulose, another major component of the biofilm matrix, is synthesized by cellulose synthase and is regulated by the bcsABZC [69]. Cellulose provides rigidity and protects from adverse environmental conditions [69]. Environmental DNA (eDNA), another biofilm component, has two layers: outer and inner, as seen in *P. aeruginosa* and *S. aureus* biofilms. The outer slime layer provides stability to the biofilm and helps transport nutrients through its pores [14],[70]. The inner layer increases antibiotic resistance by supporting the transfer of genetic information [71].

## 4. Biofilm communication

It is apparent that bacterial biofilms are pathogenic and can have detrimental effects on an individual's health in addition to resistance to certain drugs [72]. The nature of a biofilm depends on bacteria types, their strains and environmental conditions [14],[73]. Biofilms are composed of microbes, DNA and RNA, polysaccharides, proteins including enzymes and water [60],[74]. Cationic exopolysaccharides, which are suggested to be a polysaccharides rich in glucose, support the biofilm formation [75]. Pentasaccharides enhance the cross-linking between cells and between cells and the surfaces of both biotic and abiotic materials [75],[76]. Mycolic acid, on the other hand, is another vital molecule that protects the organisms in a biofilm by its immune resistance activity [77]. EPS is formed through the complex extra- and intra-cellular processes in microorganisms and plays an important role in biofilm shaping, cell communication (QS) and flow of nutrients [2],[8]. QS is extremely important in the microcolonies of the biofilm and accelerates bacterial gene expression, increasing the bacterial virulence and pathogenicity [2],[78]. It also helps with DNA transformation or plasmid transconjugation from one organism to another, facilitating and increasing the antibiotic resistance among different organisms in biofilm [79]. In addition, QS is widely known for regulating physiological activities of the bacteria through small diffusible signal molecules to increase the social interactions and biofilm pathogenicity [78]. In some bacteria, swarming motility appears to be responsible for the early development of a biofilm because of rapid surface motility [80]. Some components in EPS are exoenzymes that handle the modification, utilization and the formation of EPS layers by acting specifically on extracellular polysaccharides, proteins, lipids and eDNA [81],[82]. Not all bacteria or organisms form EPS, such as mycobacteria. They do not have the matrix structures seen in other biofilms but have other structures that are like EPS [79],[83].

## 5. Biofilm study through microsystems

Since biofilms are extremely sensitive to environmental factors, their physiologies and functions are variable, creating challenges for effective control or elimination. Microsystems have emerged to control the fluctuations of different environmental factors, hoping to minimize variance among repeated experiments. The optical, electrochemical and mechanical microsystems which are in use to characterize biofilms will be briefly discussed.

Optical microsystems have been standardized and optimized recently to study biofilms. The traditional optical microsystems, such as confocal microscopy, crystal violet staining and scanning electron microscopy (SEM), support end-point measurements of biofilms. Real time, non-invasive, non-destructive and label free optical systems have been developed to monitor the biofilm formation. For example, the modified confocal reflection microscopy (CRM) system allows the visualization of microbial growth in a biofilm over a period [84]. The dynamics of biofilms with high spatial resolution showing chemical bond signatures in biomolecules can be studied through synchrotron radiation-based Fourier transform infrared (SR-FITR) spectromicroscopy [85]. Mechanical properties of a biofilm, such as stiffness of the matrix, have been studied through the Brillouin micro-spectroscopy and Raman micro-spectroscopy [86]. In addition, biofilm physiology can be characterized by surface plasmon resonance (SPR) [87]. It is further advanced as electrochemical SPR, which can help study the formation of electroactive biofilms [88],[89].

The electrochemical biosensors are well-evaluated under three broad categories of operating principle: impedimetric (non-faradaic), potentiometric (faradaic), and amperometric (faradaic) transducers. Impedimetric transducers detect and quantify the bacteria in real time at a temperature by sensing biological samples via impedance [90]. Electrochemical impedance spectroscopy (EIS) extends the study about the adhesion properties of bacteria biofilms [91]. Measurements of concentrations of sodium and potassium ions, oxygen and pH levels within biofilms are possible through multi-electrode systems in a complex environment [92],[93]. Faradaic microsystems are useful in real-time sensing of biofilms and depend on the faradaic current generated because of the redox reaction [94]. Chemical distribution can be monitored through faradaic microsystems, which can characterize the biochemical and regulation processes in microcolonies [90],[95].

Mechanical microsystems, which usually use a thin-film piezoelectric material that can respond to electrical signals in real-time, are commonly used for biofilm sensing. Quartz crystal microbalances (QCMs), surface acoustic wave sensors and quartz tuning for oscillators are applicable to continuously monitoring the bacterial cell attachment, biofilm growth and complex cell-surface interactions [90],[96]–[98].

## 6. Biofilms in public health

Biofilms have both positive and negative impacts on public health issues [72]**.** They can be beneficial by protecting our bodies from certain harmful agents present in an environment through remediation of soil and groundwater. They are also helpful for plant protection, wastewater treatment and inhibition of corrosion [99]. However, biofilms are considered detrimental agents to our health. An example would be the formation of dental plaque, leading to serious oral and systemic health problems. Medical devices, such as pacemakers, catheters, prosthetic heart valves, contact lenses, breast implants and cerebrospinal fluid shunts, are the most common abiotic surfaces for biofilm formation. Biofilms can also grow on biotic surfaces, such as teeth, lungs and bone [2],[100]–[102]. Biofilms that grow in water systems supplying healthcare facilities are a serious problem, such as biofilms of *Pseudomonas aeruginosa* in metal water pipes [103],[104]. Such biofilms transferred to an individual are usually related to life-threatening infections, such as cystic fibrosis, periodontitis, infective endocarditis, otitis media, osteomyelitis and chronic wounds [105],[106]. Biofilms account for up to 80% of microbial infections according to the National Institutes of Health (NIH). *Staphylococcal* species are the leading cause of implantable device-associated infections [107],[108]. Biofilms play a significant role in various diseases, such as chronic respiratory infection, chronic lung disease, chronic obstructive pulmonary disease (COPD) and ventilator associated pneumonia [109],[110]. Secondary infections can sometimes cause severe bacteremia or septicemia after biofilm organisms enter into the blood through implanted devices [111],[112].

Scientists have realized the biofilm risks to public health. The health and safety issues can be divided into four categories: 1) resistance that leads to increased tolerance to most commonly used antibiotics [113], 2) persistent diseases favoring biofilm extension and modification with variable microcolonies and routinely shifted EPSs [8],[114], 3) failure of the host immune system to destroy biofilms [115] and 4) the cross contamination of clinical instruments that leads to a complex and novel biofilm depositing new and different EPS layers that protect organisms from adverse conditions [79],[116].

Biofilms are resistant to hosts' immune systems, and external stressors such as pH, osmolarity, nutrients and mechanical and shear forces [3],[36]. Resistance and survival, even in adverse conditions, help microbes colonize in various implantable materials, such as dental implants and catheters [100]. Most organisms in a biofilm are protected by the EPS layers and are resistant to various antimicrobial drugs [79],[117],[118]. The glycocalyx can trap antibacterial drugs at their maximum capacity, reducing the effectiveness of the drugs against the organisms present in a biofilm as well as plays an antimicrobial role [30],[118]. Some biofilms release enzyme-like substances that can transform certain antibacterial drugs into non-toxic materials that have no effect on the biofilm organisms [119],[120]. Certain bacteria in biofilms have metal degrading abilities that can convert active-state minerals like copper, zinc and silver to non-active states [121],[122].

## 7. Biofilm infections

Infections related to bacterial biofilms are categorized into two types: device and non-device related. Infections associated with different medical devices, such as catheters, contact lenses, pacemakers and prosthetics, are mild to severe and may include septicemia and keratitis [111],[123]. The most encountered cause of infection due to bacterial biofilm is often associated with indwelling catheters [22],[102]. Catheters used for less than 10 days have a high chance of biofilm formation on their external surfaces, while catheters used for over 30 days are more likely to develop biofilms in their lumens [2],[23]. Bacteria that grow in catheters depend on the fluid compositions administered through the catheter [22],[124]. The most common species are Gram-negative bacteria, such as *P. aeruginosa*, *Enterobacter* and *Klebsiella* species, which can easily grow in intravenous fluids [125]. The most commonly used catheter is the urinary catheter, which is inserted through the urethra into the bladder and is usually composed of latex or silicon, which favors bacterial attachment [126]. An open system catheter is more prone to bacterial contamination and, therefore, transfers of diseases, such as urinary tract infections (UTIs). *E. coli*, *Enterococcus faecalis* and *Proteus mirabilis* are among the most common pathogens in UTIs [127]. Contact lenses are also susceptible to bacterial biofilm growth because of the adhesive nature of both the lenses and the containers used to keep cleaning lens solution [128]. Bacteria, such *as E. coli*, *S. aureus*, *Serratia* and *Proteus*, are responsible for biofilm formation in contact lenses and cause severe infections, such as keratitis. In addition, *P. aeruginosa* may lead to blindness following severe keratitis [128],[129]. Other organisms, such as *Streptococcus*, *Enterococcus* and *Candida*, can form biofilms on mechanical heart valves and cause endocarditis [130],[131].

Non-device related diseases associated with biofilms occur when our own organs provide suitable biotic surfaces to organisms for attachment and support for the release of extracellular proteins which form EPS. For example, *Fusobacterium nucleatum* and *P. aerobicus* infect the gingiva and cause periodontitis when oral hygiene is poor [132],[133]. The biofilms established on the tooth surface interfere with the flow of calcium in the epithelial cells, ultimately forming plaque or tartar, which is a mineralized biofilm made of mainly calcium and phosphate ions [132],[134]. Osteomyelitis is another example of non-device related disease that can be caused by biofilms transported to the bone metaphysis through the bloodstream [135]. Immune reactions to the microorganism further deteriorate the bone tissue, leading to fracture [135]–[137]. In addition, the biofilms formed in open wounds of diabetic patients lead to chronic disease. Anaerobic bacteria colonize the interior while aerobic bacteria forms biofilms at the surfaces of deep wounds [138],[139].

Various studies have shown that biofilms are often responsible for chronic infections [72],[140],[141]. *S. aureus* and *P. aeruginosa* are most common biofilm organisms for chronic diseases in diabetic patients. Biofilms may delay treatment for years due to antibiotic resistance [139],[142]. Interestingly, the abnormal collection of water in some internal organs supports the reduction of tissue viscosity and accelerates biofilm formation [143]. For example, *P. aeruginosa* biofilms can cause serious lung infection in patients with cystic fibrosis (CF) that is often difficult to treat [144],[145]. *Staphylococci, Streptococci*, Gram-negative bacteria, and some fungi can form biofilms in intravenous catheters, and cause prosthetic valve endocarditis (PVE) and native valve endocarditis (NVE) in recipients. Both PVE and NVE can further extend to non-bacterial immune-related thrombotic endocarditis [72]. Coagulase-negative *Staphylococcus* (CoNS) can cause endocarditis, either by its circulation to the heart from local chronic wounds or by its direct transmission to the heart by contaminated cardiac valve prostheses [146]. Various Gram-positive and Gram-negative microorganisms are constant occupants in beds and often produce chronic polymicrobial infections, such as bed sores [147]. A few clinical circumstances exemplify the concern that biofilms resistant to pro-inflammatory reactions are responsible for severe diseases [148],[149]. The presence of microbial biofilms, which may go unnoticed during diagnosis and treatment phase, can lead to misdiagnosis and severe health consequences [140],[150].

## 8. Biofilm drug resistance

Diseases caused by microbial biofilms are serious public health issues because of the increasing antimicrobial resistance. It has been demonstrated that bacteria in biofilms are a thousand times more resistant to antibacterial drugs compared to those in the planktonic state [151]. The capsule-like outer EPS layer increases the resilience of organisms by restricting the entry of various antibacterial drugs, in addition to managing various regulatory systems. EPS can expel the harmful molecules out instead of allowing their entry into the biofilms according to the study by Mosaddad *et al*. [152]. It also acts as a barrier not allowing polar charged antibiotics [119]. The physiological components of a biofilm, such as nutrients and oxygen circulating through water channels in the EPS, also contribute to drug resistance [79],[120]. Over time, biofilms grow in size and shape, leading to the development of an interior anaerobic environment with minimal supply of nutrients and other molecules [42],[54]. Some antibiotics, such as β-lactams, fluoroquinolones and aminoglycosides, are ineffective to the inner anaerobic biofilm since they are inactive in the absence of oxygen and nutrients [153],[154]. Biofilms support gene transfer among microorganisms through their various connection channels, developing antibiotic resistance [118],[155].

Biofilms are structurally and dynamically complex, and they are resistant to common antibiotics [156]. In addition, biofilms have multiple tolerance mechanisms, such as impermeability, rapid growth, differential nutrient gradients and gene transfer [155]. Antibiotic penetration into biofilms primarily relies on the EPS structure that can confer impermeability to large molecules of aminoglycosides [52],[79]. In addition, EPS can quench antibiotic activities through certain mechanisms, such as diffusion-reaction inhibition and enzymatic degradation pathways [52],[157]. Even though EPS composition varies among the different biofilms, it usually contains lipopolysaccharide and alginate, both acting together as a barrier for antibacterial drug diffusion [155],[158]. Increased alginate in the EPS of the biofilm made of a mixed variant of *P. aeruginosa* helps overcome the immune system, ultimately extending the infection to the more chronic disease state [42],[159]. Mannuronic acid and guluronic acid residues present in the EPS of some biofilms act as potential virulence factors to prolong infections by suppressing immune responses and by protecting the biofilms from antibiotics, such as ciprofloxacin, gentamicin and ceftazidime.

Over time, bacteria can adapt to new adverse environments after horizontal gene transfer among microcolonies with no changes in biofilm [160]. *P. aeruginosa* is one of the bacteria that possesses a high level of antibiotic resistance. Because of its capacity for frequent spontaneous mutation, it can increase the production of β-lactamase, inactivating the β-lactam antibiotics [161]. Mutation also facilitates adjacent microcolonies in a biofilm taking up free DNA, making more antibiotic resistant biofilms [71]. eDNA increases biofilm resistance by two different mechanisms: 1) changing the outer membrane composition of the biofilm and 2) chelating cations, such as calcium and magnesium, as seen in *P. aeruginosa, S. aureus, Enterococcus faecium, Klebsiella pneumoniae, Acinetobacter baumannii, Enterobacter* species and *Salmonella enterica* [162],[163]. All pathogens are responsible for drug-resistant biofilm associated infections.

*E. faecium* causes biofilm mediated infections in patients implanted with medical devices. It releases eDNA for biofilm attachment and stability by autolysis of its subpopulation through the QS system, similar to *S. aureus* and *S. epidermidis*. This process is called fratricide and is mediated by autolysis or murein hydrolase [164],[165]. Low permeability of its cell wall to large molecule aminoglycosides and overexpression of low-affinity penicillin-binding proteins are responsible for antibacterial resistance in *E. faecium* to aminoglycosides and β-lactam antibiotics, respectively [166]. Rearrangement of the adherent biofilm cells in the monolayers because of genetic alternation in the presence of antibiotics-induced stress also leads to higher resistance [167]. Cell heterogeneity with different QS expression patterns with antibiotic persister cells in a biofilm of *S. aureus* leads to cell survival in the presence of antibiotics without development of resistance [168],[169]. Antibiotic persister *S. aureus* has low metabolic activity producing less ATP in the presence of low oxygen and nutrition, which eventually contributes to elevated antibiotic tolerance in biofilm [170]. Certain enzymes, such as proteases and nucleases produced by *S. aureus*, disperse in the biofilm, enhancing the survivability of the biofilm cells [171].

*K. pneumoniae* produces a thick layer of extracellular biofilm on both biotic and abiotic surfaces, increasing antibiotic tolerance [172]. Virulence factors, such as polysaccharides, lipopolysaccharides, fimbriae and outer membrane proteins participate in the biofilm formation [173]. *K. pneumoniae* is a greater biofilm producer than *Enterobacter*, which is moderate biofilm producer *in vitro* [174]. Similar to *K*. *pneumoniae*, *A. baumannii* in biofilm is more antibiotic resistant than *Enterobacter* and causes nosocomial infection after its entry through vascular catheters or Foley catheters. It adheres to the host cells with the help of fibronectin [175],[176]. The surface protein, AbOmpA, is present in the wall surface of *A. baumannii*. It is responsible for the pore formation in eukaryotic cells, supporting adhesion and biofilm formation along with other adhesion proteins and extracellular polymeric compounds [177]. The pathogens that cause nosocomial infections in hospitals usually form the biofilms which are resistant to multi-drug antibiotics. Among Gram-negative bacteria, *E. coli* (38%) is a predominant clinical isolate that forms biofilms, along with *Acinetobacter* species (20%), *Klebsiella* species (16%) and *Pseudomonas* species (12%) [178].

Metabolically active organisms release more enzymes and enhance the biofilm growth, influencing drug resistance, while slow-growing organisms have minimal or negligible concentrations of enzymes without affecting drug resistance [179]–[181]. The metabolically active organisms are, however, more susceptible to antibiotics compared to the less active organisms. For example, slow-growing organisms are less susceptible to antibiotics with more efficacy in drug resistance [182]. Most sessile organisms with negligible metabolic activity are categorized as persisters. Unlike mutants, they are at the state of no growth and represent a tiny part of the population with high resistance to antibiotics [156]. Some aerobic organisms, such as *P. aeruginosa*, become resistant to antibiotics (e.g., ciprofloxacin and tobramycin) when less oxygen is available in the microenvironment [183],[184].

Biofilm resistance and tolerance to antibiotics created by anti-penetration properties of the matrix, presence of polysaccharides, antibiotic-modifying enzymes, eDNA and bacteriophages have been well studied by Hall and Mah [185]. They also mentioned that physiological heterogeneity and hypoxic conditions increase the antibacterial resistance [185]. The heterogeneity creates a nutritional deficiency for certain bacterial populations, which favors more resistance [186]. Biofilm heterogeneity with both genotype and phenotype is one of the most important factors that provide resistance to the bacterial communities in biofilms against many antibacterial drugs [185],[187]. Antibiotic resistance in biofilms can be transferred among the biofilm organisms through external mechanisms such as QS and EPS. They can regulate the bacteria behavior through signaling cascades and gene expression [188],[189].

## 9. Biofilm control strategies

**Figure 3.**
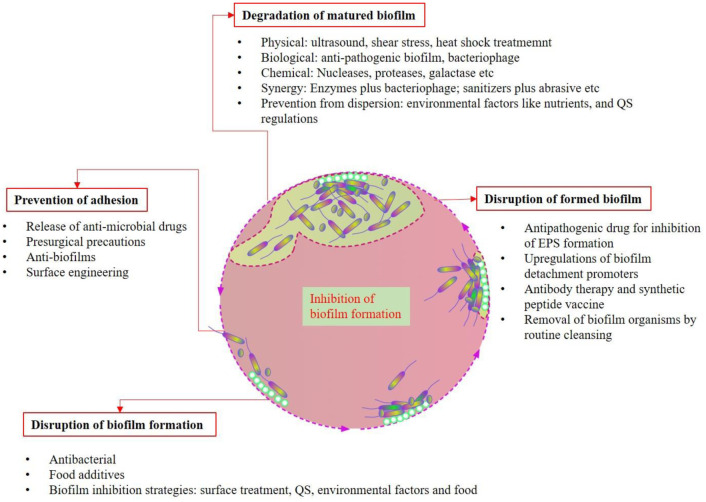
Inhibition of biofilm formation.

Finding an effective way to control biofilm related diseases is a challenge, but failure to do so will only increase mortality and morbidity rates [190]. Developing new strategies to control biofilms is important for healthcare facilities because of the misuse and overuse of antibiotics, favoring biofilms overcoming the current antibiotics and leading to recurrent infections [191]. In-depth knowledge and understanding of the mechanisms of biofilm matrix formation, its structural components and how communication occurs aid in developing better methods to impede colonization on medical or non-medical devices [69]. Constructing future novel alternative antibiotics or even vaccines targeting these areas can only minimize biofilm long-term infection [2]. Previous studies have demonstrated that combined traditional antibiotics have stronger antibiofilm potency [190]. Strategies depend on the stage of the biofilm, from its attachment to its dispersion, as shown in Figures 1 and 3. Prevention of bacterial adhesion can be accomplished through various strategies, such as coating antimicrobial drugs on implants, presurgical precautions and surface engineering that inhibits the microorganism attachment [192],[193]. Biofilm formation can be disrupted in the initial stage by various antibacterial molecules, food additives and surface treatment, in addition to alterations in other various environmental factors [119],[194] (Figure 3). Elimination of established biofilms is relatively challenging, but there are many approaches, such as EPS inhibitors, detachment promoters, vaccine therapy and mechanical removal of visible biofilms [195]–[198] (Figure 3). Degradation of mature biofilms through various physical, biological, chemical and synergistic methods has been applied in clinical practice [120],[199] (Figure 3).

Biofilm formation can be inhibited at any stage of biofilm formation, as described in Figure 3. Bacterial adhesion can be prevented by various approaches, such as release of antimicrobial drugs, presurgical precautions, anti-biofilms and surface engineering. Biofilm formation can be disrupted by using various antibacterial agents, food additives, surface treatments and alterations in environmental factors, etc. Disruption of formed biofilm is challenging compared to that of the immature biofilm. Different strategies have been applied for this purpose: for example, inhibition of EPS formation by application of antipathogenic drugs, upregulation of biofilm detachment promoters, antibody therapy and use of synthetic peptide vaccine and removal of biofilm organisms by routine cleansing procedures. There are various methods that can be applied for the degradation of matured biofilm: for example, physical (e.g., ultrasound, shear stress, heat shock treatment), biological (e.g., anti-pathogenic biofilm, bacteriophage), chemical (e.g., application of enzymes, such as nucleases, proteases and galactase) and synergistic approaches (e.g., enzymes plus bacteriophages and sanitizers plus abrasives) and prevention from dispersal (e.g., application of environmental factors to dysregulate nutrients and QS system).

## 10. Conventional antibiotics

**Figure 4.**
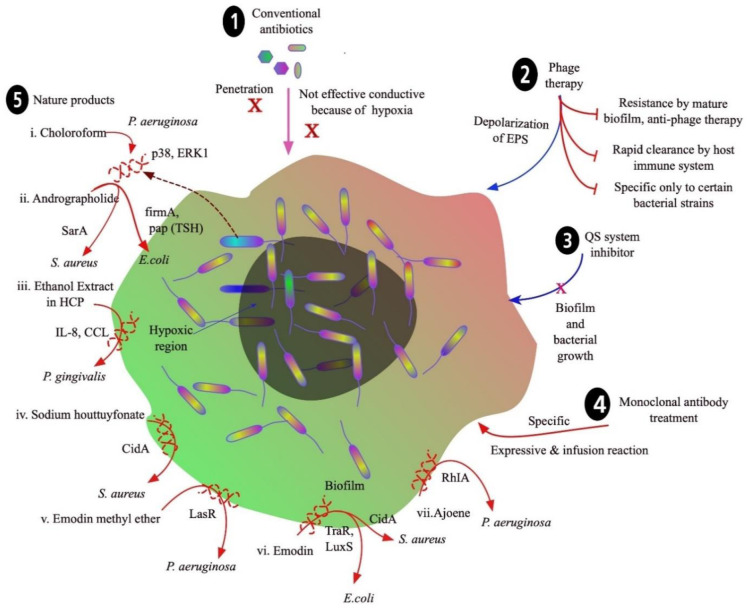
Conventional and new approaches to control biofilm formation.

The failure of classic antibiotic therapies has led to worsening situations globally, such as antibiotic resistance of biofilms in medical implants and catheters. Biofilms dynamically modify their EPS, which makes them impervious to various antibiotics (Figure 4). Orally administered β-lactams, quinolones, aminoglycosides and macrolide antibiotics are prime examples of treatment failures. β-lactams are hydrolyzed by bacterial β-lactamases, so they are ineffective and cannot penetrate biofilms [200]. Quinolones, aminoglycosides and macrolides are transported out of bacterial cells by efflux pumps [201]. In addition, quinolones are not more effective in the anaerobic environment of the biofilm [202]. Although difficult to treat with classic antibiotic therapy, biofilms have shown susceptibility to certain classes of drugs, when delivered correctly and in high doses. To treat these infections, many combination therapies, such as high dose topical treatments or antibiotic adjuvants, have been used [202]. Cephalosporins, aminoglycosides, monobactams, polymyxins, tetracyclines and glycylglycines have been proven to be effective against biofilms through topical administration in higher doses [203]. Biofilm infections in the lungs have been treated successfully through the inhalation of colistin, tobramycin, aztreonam, ciprofloxacin, levofloxacin and gentamicin. Some antibiotics, such as vancomycin, minocycline, linezolid, daptomycin, tigecycline, rifampicin and cephalosporins, applied directly to tubing or medical devices have been shown to prevent biofilm formation [202],[204].

## 11. Alternatives to conventional antibiotics

Many new methods of prevention and treatment are being studied and developed because of biofilm resistance to conventional antibiotics. There are various approaches to developing novel antibiofilm agents, such as small molecules capable of inhibiting some virulence factors, and enzymes targeting the matrix. DNase, proteinase K and trypsin are some enzymes that target matrix proteins and degrade eDNA, decreasing the rigidity and stability of the biofilm, as seen in *S. epidermidis* biofilm [119],[205]. Benzimidazole and N-acetylcysteine are chemicals that inhibit synthesis of EPS and support biofilm degradation [206],[207]. Microbial adherence can be inhibited by using certain chelators that bind ions, such as calcium and magnesium, preventing bacterial attachment to surfaces [208]. Bactericidal or bacteriostatic agents are used to coat the surfaces of medical devices. For example, metal implants are coated with broad-spectrum antibiotics, such as vancomycin, to inhibit biofilm formation. Studies showed effective results in inhibiting *S. epidermidis* biofilms, but some reports show that bacteria could develop resistance to vancomycin [209],[210]. Some bactericidal agents, such as silver, may inhibit biofilm formation by damaging DNA and microbial proteins [211]. Even though coating medical devices with silver nanoparticles showed good results, the use of high doses can also damage human cells because of their toxic effects [212]. The bio-inspired quercetin nanoparticles act as anti-adhesive agents against certain bacteria (e.g., *Bacillus subtilis*), preventing surface attachment [213]. Another coating antimicrobial agent is furanone, which shows good results in inhibiting biofilm formation by *S. epidermidis* [214]. Devices made of silicone rubber are usually coated by trimethoxysilyl propyldimethyloctadecyl ammonium chloride (QAS-30), which has bactericidal and bacteriostatic effects through its quaternary ammonium groups [215]. Another approach for inhibiting biofilm formation in medical devices and prosthetics is the repulsion of microbes from surfaces, which is accomplished by applying an anti-adhesion layer. The coating creates a rough texture or a charged surface, making it less compatible to proteins [216]. Some examples of these coating compounds are trimethylsilane (TMS), selenocyanatodiacetic acid (SCAA), and polymer brush [217]. Medical devices, such as implants, made of stainless steel and titanium coated with TMS showed significant inhibition of microbial biofilms, especially those produced by *S. epidermidis*. SCAA is an organo-selenium compound that releases superoxide radicals and inhibits biofilm formation in coated surfaces of hemodialysis catheters [218]. Another type of anti-adhesion is polymer brush coating made of polyethylene oxide (PEO), which creates a repulsive osmotic pressure to repel bacteria and proteins. This reduces microbial adhesion *in vitro*, but it shows no effect *in vivo* because of its weak attachable capacity to the moisturized medical devices since microbial flagella and pili help movement and adhesion [217]. Most opportunistic organisms that form biofilms can cause infection, but some actually protect against more infectious pathogens [219].

The novel antibiofilm agents are composed of both natural and synthetic molecules, which include halimane diterpenoids, imidazole and indole derivatives, plant extracts, peptides and polysaccharides [42]. Several degrading enzymes, drug delivery nanoparticles and cell-damaging photodynamic therapy (PDT) have also been recently investigated in treating biofilms [79]. Perhaps the most pervasive natural antibiofilm agents are halimane diterpenoids, which can be found in terrestrial plants, marine organisms and bacteria. They are versatile compounds and are used industrially for agricultural, pharmaceutical and beauty products, with their ability for antibacterial and antimycobacterial effects [220],[221]. 2-aminoimidazole, a natural imidazole derivative, has also been used to prevent and dissipate the biofilms formed by methicillin-resistant *S. aureus* (MRSA), *A. baumannii, P. aeruginosa, V. cholerae, K. pneumonia*, and *Shigella boydii*. Besides imidazole derivatives, indole derivatives have been shown to be effective against biofilms by affecting bacterial cell signaling and gene expression [42]. Interestingly, various natural plant extracts, including garlic, hordenine, limonoids and quercetin, are effective against biofilms by inhibiting the transcription of certain genes required for QS. Certain products, such as cranberry polyphenols, inhibit the production of virulence factors that are required for bacterial adhesion and bacterial structures needed for the formation of biofilms [222]. An antibiofilm peptide produced by the human body, called LL-37, stops bacteria from attaching and forming biofilms on mucosal surfaces. LL-37 has been shown to be effective against strains of *P. aeruginosa*, group A *Streptococcus* (GAS) and *S. epidermidis*. Larger polysaccharide molecules, such as galactan and galactose, can also have effects on biofilm formation. Galactan is produced by *Kingella kingae* and prevents other species of bacteria from forming biofilms [42],[223]. Ethylcholine, one of the novel cholines, inhibits biofilm formation without decreasing bacterial growth, as seen in *P. aeruginosa* [224]. Some other alternate products are outlined in the Figure 4.

Because of increasing biofilm resistance against currently available antibiotics, different new approaches have been studied, as shown in Figure 4. Conventional methods (1) usually contain antibiotics. Biofilms become more resistant because of the failure of most antibiotics to penetrate the biofilm EPS, and another reason could be a hypoxic environment that hinders effective distribution of antibiotics throughout the biofilm. Phage therapy (2) is another conventional method that disrupts the biofilm and its formation by depolarization of EPS; however, biofilms become resistant against phage therapy. Certain mature biofilms are resistant to phage therapy, and bacteria in biofilms can release anti-phage substances that neutralize the phage. In addition, the immune system clears the phage proteins rapidly from the body, so it is less effective on the biofilm. Another important limitation of phage therapy is that it is more specific to certain bacterial strains, so it is not applicable to other biofilm types. New, emerging strategies have been applied to disrupt biofilm. For example, QS system inhibitors (3), monoclonal antibody treatment (4) and use of nature products (5) are being studied extensively to control and eliminate biofilm. New nature products are under evaluation for their efficacy in eliminating the bacterial biofilms which are more resistant to commonly used broad-spectrum antibiotics. For example, chloroform, andrographolide, ethanol extract of *Houttuynia cordata* poultice (HCP), sodium houttuyfonate, emodin methyl ether, emodin and ajoene inhibit biofilm formation of different bacteria, such as *P. aeruginosa* and *S. aureus*, by suppressing various factors that are responsible for bacterial survival and virulence, as shown in Figure 4.

## 12. Micromolecules

Understanding microorganisms and their biofilm formation mechanisms might lead to the development of certain micromolecules. Such a strategy has been studied by dentists through the process of cavity formation by *Streptococcus sobrinus* and *S. mutans* [225]. This strategy has also been used in other fields to minimize the cases of biofilm infection. However, the rise of drug-resistant bacteria has led to the reassessment of the bacterial mechanisms and new developments by genetic alterations [196],[226].

## 13. Phage therapy

Phage therapy has been used as an alternate approach for controlling biofilm formation. It works by depolarizing the EPS, leading to the disruption of the biofilm. Mero *et al*. and Dickey *et al*. have described phage therapy as an alternative strategy to control biofilm [227],[228]. This method has certain limitations, including resistance by mature biofilms, rapid clearance by the host immune system, ineffectiveness against certain bacterial strains and fast development of phage-resistant subpopulations within a short time period [228]–[230] (Figure 4).

## 14. QS system inhibitors

QS is a microbial communication mechanism based on molecular signatures and is responsible for the regulation of biological functions, including the production of virulence factors and formation of biofilms [231] (Figure 4). QS system inhibitors are effective in inhibiting regulatory genes and virulence factors [232]. Commercially available anti-QS compounds could increase bacterial biofilm susceptibility to antibiotics both in vivo and in vitro, as demonstrated by Brackman *et al*. [233]. QS-inhibitors, such as *N*-(4-[4-fluoroanilno]butanoyl)-L-homoserine lactone (FABHL) and *N*-(4-[4-chlororoanilno]butanoyl)-L-homoserine lactone (CABHL), can effectively disable the QS system of *Pseudomonas aeruginosa* by down-regulating the expression levels of lasR and rhlR genes [234]. QS-inhibitors increase the efficacy of certain drugs by acting synergistically to control biofilm formation. This has been demonstrated by experiments with polypeptides [235], cephalosporins [236], aminoglycosides [237] and quinolones [238].

## 15. Monoclonal antibody treatment

Antibody-based antibiofilm therapy has been implemented and has shown promise in preclinical models that target several components present in biofilms. However, this method has been limited by target specificity and infusion reactions [239] (Figure 4). Pooled monoclonal antibody treatment significantly decreases biofilm formation and demonstrates promising therapeutic effects to prevent biofilm infection. For example, monoclonal antibodies 12C6, 12A1 and 3C1 were used against *S. epidermidis*, where they specifically bind to the bacterial accumulation-associated protein (AAP) to inhibit the biofilm formation [240]. A native human monoclonal antibody, TRL1068, binds to the DNABII proteins from both Gram-positive and Gram-negative bacteria and disturbs the biofilms of *S. aureus* and *P*. *aeruginosa* [241]. Together with antibiotics, TRL1068 would support the control of biofilm formation [241]. Monoclonal antibodies target specific antigens, such as adhesin proteins (ClFA, FnBPA, Can, SasG), to disrupt the formation of biofilms [242]–[245]. They also bind specifically with cell wall-modifying enzymes (Atl, Atl-Amd, Atl-Gmd, IsaA) and inactivate them to inhibit dynamic changes in the EPS [246]–[249]. Monoclonal antibodies against glycopolymers (WTA, CP and LTA) have been shown to promote opsonophagocytic killing (OPK) effects [250],[251]. Anti-matrix component antibodies such as PNAG and DNABII, immune evasion proteins such as Spa, toxins such as HIa and LukAB and proteins such as PhnD have also been studied [252].

## 16. Natural products

Biofilm formation by multi-drug resistant microorganisms is a major concern faced by clinicians and scientists in establishing potential antibacterial therapeutics. Several bioactive compounds exhibiting antibacterial activity have been extracted, purified and subjected to clinical testing to check their efficacy in inhibiting biofilms [253]. Natural products, such as chloroform extract, andrographolide, ethanol extract, sodium houttuyfonate, emodin methyl ether and emodin, inhibit the biofilms through various mechanisms that target bacterial cell signatures, as illustrated in figure 4. Chloroform extract of *Andrographis paniculata* has shown a significant reduction of the QS-controlled extracellular virulence factors in *P. aeruginosa* infections. It also has the potential to inhibit swarming motility and biofilm formation by decreasing p38 and ERK expression levels in the MAPK signal pathway [254] (Figure 4). Andrographolide disrupts the biofilm of *S. aureus* by inhibiting the transcriptional factor SarA and also works against *E*. *coli* by inhibiting fimA and pap (TSH) [255],[256]. In addition, natural product extracts in ethanol have been widely used for the control and elimination of anaerobic biofilms, such as in the suppression of *P*. *gingivalis* through the inhibition of IL-8 and CCL [257],[258]. Sodium houttuyfonate obtained from the oil of the plant *Houttuynia cordata* has been used for many years in clinical practices as an antimicrobial agent. It has been found to inhibit the biofilms of *S. aureus* and *S. pneumoniae* by suppressing the transcription levels of autolysis cidA [259]. Natural medicines, such as emodin and ajoene, have been shown to control the biofilms of various microorganisms (e.g., *P. aeruginosa, E. coli* and *S. aureus*) [260]–[263].

## 17. Probiotics

**Figure 5.**
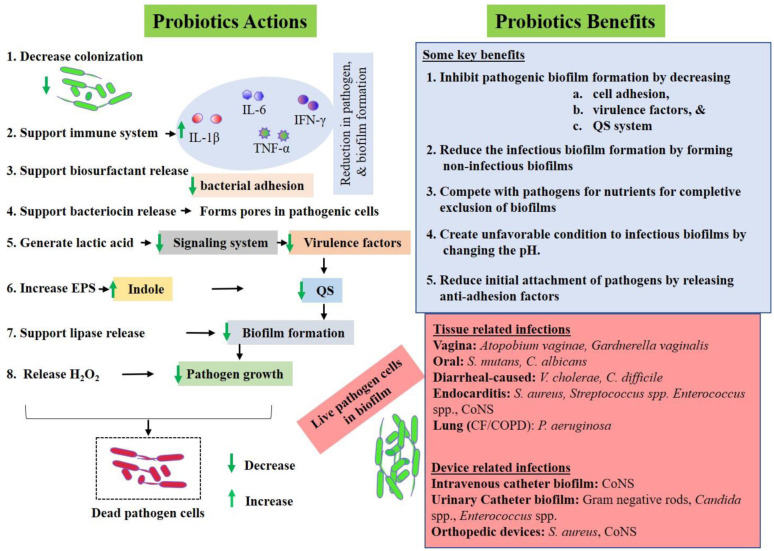
Probiotic approaches to control biofilm formation.

Probiotics are live microorganisms that confer health benefits to the host when they are administered in adequate amounts. Microorganisms, which show inhibitory activities against some pathogens, adherence to epithelial cells and resistance to certain concentrations of bile and acid, are usually considered as probiotics [264],[265]. In addition, they should be able to withstand antibiotics and bind to pathogens sufficiently to inactivate them [266]. Due to increased antibiotic resistance, probiotics that can inhibit biofilm formation have garnered attention. *E. faecium* WB2000, *Bifidobacterium* BB12 and *Bifidobacterium adolescentis* SPM1005 are some probiotics that have been considered for their role in inhibiting bacterial biofilm formation [267]–[269]. As shown in Figure 5, probiotics provide different molecules that help to suppress colonization [270],[271], reduce bacterial adhesion [271], boost the immune system [272], release bacteriocin to inhibit bacterial growth [273], maintain lactic acid in the environment that reduces bacterial virulence signaling systems [274], suppress EPS from pathogens [275] and increase the indole in the biofilm which down-regulates the QS system [276] and lipase inhibitor production [277]. Products of probiotics, such as bacteriocin, can lyse the pathogens by making pores into their cell walls [278]. In addition, hydrogen peroxide (H_2_O_2_) might be harmful to certain pathogenic microcolonies within the biofilm [279]. Probiotics, therefore, may play a vital role in inhibiting pathogenic biofilm from its initial stage of attachment and development to its dispersion. This occurs through various mechanisms, such as anti-adhesion activities, QS system suppressors and formation of non-infectious biofilms, that can compete with the nutrients and pH change, as illustrated in Figure 5 [280]–[282]. Probiotics can act as anti-pathogenic biofilms in both tissue-related and device-related infections [283]–[285].

Probiotics support pathogenic biofilm inhibition in various ways, as shown in the figure. Their colonization and release of biosurfactants and bacteriocin supports the inhibition of pathogenic bacteria adhesion and growth. Probiotics produce certain acids, such as lactic acid, that alter the pH in the biofilm microenvironment, suppressing various bacterial signaling systems which are responsible for the QS system, virulence and EPS secretion. In addition, probiotics signal host immune systems to release various inflammatory factors (e.g. IL-1B, IL-6, TNF, interferons) which help to inhibit bacterial growth and biofilm formation. Broadly, probiotics inhibit biofilm formation through various ways, such as reducing virulence of pathogenic bacteria, suppressing QS systems, producing anti-adhesion substances and altering biofilm optimal microenvironment by changing pH, nutrition and oxygen gradient, as shown in the figure. Probiotics would be effective for both tissue and device-related infections caused by various pathogenic biofilms. CoNS: Coagulase negative *Staphylococci*.

## 18. Gene editing techniques

Genetic alterations on biofilm pathogens might be possible through gene editing tools like short palindromic repeat-CRISPR- associated (CRISPR-Cas) systems, which could make them less virulent over time [286],[287]. CRISPR-Cas has been used in the engineering of bacterial genetics and the reversal of antibiotic resistance by targeting appropriate genes [288],[289]. The details of CRISPR-Cas gene editing techniques are beyond the scope of this review paper. This technique has been applied to minimize the resistance to various antibacterial drugs carried by plasmids of some infectious bacteria, such as *E. coli*, and also to target the specific genes in virulence and antibiotic resistance in bacterial populations [290]–[292]. Further, the CRISPR-Cas system has been used to neutralize the bacterial genes responsible for antibiotic resistance [291],[293]. Gene editing techniques are being developed to deliver polymeric nanoparticles effectively and target the virulent gene efficiently, with or without combination with other delivery systems, such as phage delivery or conjugative delivery [294]. Recently, anti-biofilm applications of gene editing techniques have been studied, such as by Zuberi *et al*. to inhibit *E. coli* biofilm formation [295], by Tang *et al*. to inhibit *S. mutans* biofilm formation [296], and by Garrido *et al*. to eliminate *S. aureus* biofilms in vivo [297].

## 19. Conclusion and future prospects

A less virulent pathogen becomes more virulent, acquiring more antibacterial resistance when it is part of a biofilm. Genetic transfer in a biofilm community modifies the bacterial population, enhancing secretion of various secretory substances and changing cell signatures, which increases the bacterial resistance. Treatment failure is due to acquired resistance through various secretory substances and cell signatures. The spread of multi-drug resistant microorganisms found in biofilms is worsening situations all around the globe and emerging as a new threat to public health. We have discussed how biofilms become more virulent, the roles of various factors responsible for biofilm formation, chronic infections related to biofilms, new strategies that are promising for treating the pathogenic biofilms and various other alternatives such as, natural medicines and probiotics, that act synergistically with antibacterial drugs to combat spreading biofilm infections. In addition, other alternative approaches, such as highly diffusible nano-antibodies or micromolecule formulations, novel anti-pathogenic biofilm agents and CRISPR gene editing technologies, might be future options to treat the multi-drug resistant biofilms. Furthermore, the development of microsystems that mimic the aerobic and anaerobic biofilm microenvironment could support microanalysis of environmental factors, such as pH, nutrients, temperatures and metabolites, that play roles in microbial sensing, virulence and resistance. Controlled guidance systems from microsystems would help to formulate new drugs and set a dose to eliminate the infectious biofilms effectively from both biotic and abiotic substrates.
